# Mining Alzheimer’s disease clinical data: reducing effects of natural aging for predicting progression and identifying subtypes

**DOI:** 10.3389/fnins.2024.1388391

**Published:** 2024-08-14

**Authors:** Tian Han, Yunhua Peng, Ying Du, Yunbo Li, Ying Wang, Wentong Sun, Lanxin Cui, Qinke Peng

**Affiliations:** ^1^Systems Engineering Institute, School of Automation, Xi’an Jiaotong University, Xi’an, China; ^2^Center for Mitochondrial Biology and Medicine, School of Life Science and Technology, Xi’an Jiaotong University, Xi’an, China; ^3^The Key Laboratory of Biomedical Information Engineering of Ministry of Education, Xi’an Jiaotong University, Xi’an, China; ^4^Department of Neurology, Tangdu Hospital, Fourth Military Medical University, Xi’an, China; ^5^Department of Nuclear Medicine, Tangdu Hospital, Fourth Military Medical University, Xi’an, China; ^6^School of Future Technology, Xi’an Jiaotong University, Xi’an, China

**Keywords:** time-series analysis, T-cPCA, AD progression prediction, AD subtype identification, natural aging

## Abstract

**Introduction:**

Because Alzheimer’s disease (AD) has significant heterogeneity in encephalatrophy and clinical manifestations, AD research faces two critical challenges: eliminating the impact of natural aging and extracting valuable clinical data for patients with AD.

**Methods:**

This study attempted to address these challenges by developing a novel machine-learning model called tensorized contrastive principal component analysis (T-cPCA). The objectives of this study were to predict AD progression and identify clinical subtypes while minimizing the influence of natural aging.

**Results:**

We leveraged a clinical variable space of 872 features, including almost all AD clinical examinations, which is the most comprehensive AD feature description in current research. T-cPCA yielded the highest accuracy in predicting AD progression by effectively minimizing the confounding effects of natural aging.

**Discussion:**

The representative features and pathogenic circuits of the four primary AD clinical subtypes were discovered. Confirmed by clinical doctors in Tangdu Hospital, the plaques (18F-AV45) distribution of typical patients in the four clinical subtypes are consistent with representative brain regions found in four AD subtypes, which further offers novel insights into the underlying mechanisms of AD pathogenesis.

## 1 Introduction

Aging and age-related chronic diseases in the elderly, including age-associated memory impairment, mild cognitive impairment (MCI), and Alzheimer’s disease (AD), belong to the aging syndrome category (Fried, 2012). It is traditionally understood that AD is the result of a transformation from quantitative to qualitative changes within the normal aging process, indicating a close correlation between AD and normal aging in terms of clinical symptoms (Petersen, 2000). For instance, as an intermediate stage between normal aging and dementia, MCI is easily mistaken for natural aging because the pathological changes presented by patients in the early stages are not obvious, which makes it difficult to achieve precise treatment in the early stages of the disease (Platero and Tobar, 2020). Moreover, aging of the human body may lead to memory loss and a decline in brain function, which are considered essential factors that affect AD diagnosis (Tranah et al., 2012). Consequently, accurately distinguishing the specific symptoms of AD from those associated with natural aging in the early stages is essential to enhance the effectiveness of AD diagnosis and improve the cure rate.

Various biomarkers and clinical symptoms have been employed in AD correlational research, such as evaluating AD progression and identifying AD subtypes based on neuroimaging and biological detection technology. The pathophysiological changes in AD mainly include amyloid deposition, neurofibrillary tangles, and neurodegeneration (Yang et al., 2022). Nonetheless, existing biomarkers lack a longitudinal perspective and simultaneously combine the effects of natural aging owing to the complex neurodegenerative pathogenesis (Franzmeier et al., 2020). Additionally, there is no consensus on the most effective biomarker for early diagnosis because each biomarker differs in terms of sensitivity, specificity, and reliability. For instance, almost one-third of clinically diagnosed AD patients do not have an accumulation of Αβ in specific brain regions, such as the hippocampus and frontal lobe, and many people who had Αβ accumulation after death did not show cognitive impairment during their lifetime (Sekiya et al., 2018). Therefore, the extraction of more useful features with minimal interference from natural aging for subsequent AD analyses is a major challenge.

Research on AD has focused on featuring engineering based on extensive diagnostic data, driven by the high heterogeneity of disease progression among patients. In the early 21st century, significant advancements in imaging diagnosis technology and AD pathology research have led to the emergence of many AD progression prediction methods based on neuroimaging (Sarica et al., 2017), which focus on dimension reduction (Duchesne et al., 2009; Zhu et al., 2013) and feature selection techniques (Li et al., 2013, 2015). With the improvement of imaging diagnosis technology and the development of AD pathology research, a large number of AD process prediction methods based on Neuroimaging appeared at the beginning of the 21st century (Zhu et al., 2014; Hyun et al., 2016). Chen et al. presented a model named Low-rank Sparse Feature Selection with Incomplete Labels (LSFSIL) for predicting cognitive performance and identifying informative neuroimaging markers with MRI data (Chen et al., 2022). Lu et al. proposed a novel method to learn an enriched representation for imaging biomarkers (Lu et al., 2021). Jiang et al. proposed a novel multi-task learning formulation, which considers a correlation-aware sparse and low-rank constrained regularization, for accurately predicting the cognitive scores and identifying the most predictive biomarkers (Jiang et al., 2018). Since deep learning algorithm can mine the potential features in image data, the method of image analysis using deep learning has become the main research direction of this problem (Lin et al., 2018; Jo et al., 2019; Abrol et al., 2020). Liu et al. proposed an ensemble learning framework based on artificial neural networks to create effective models for AD/MCI prediction from multiple modalities of neuroimaging and multiple baseline estimators (Liu et al., 2016). Hojjati et al. utilized unimodal/bimodal neuroimaging measures and a non-linear regression method (based on artificial neural networks) to predict the neuropsychological scores (Hojjati et al., 2022). Hoang et al. proposed Vision Transformers (ViT) to make an MCI-to-AD prediction based on structural magnetic resonance images (Hoang et al., 2023). However, subsequent studies have revealed that AD is a heterogeneous disease influenced by diverse pathophysiological mechanisms beyond conventional understanding (Neff et al., 2021). Neuroimaging biomarkers only represent a portion of clinical manifestations of AD. Other critical indicators, such as cognitive evaluations, remain underutilized in subsequent analyses, thus failing to fully capture the development of AD.

As a longitudinal multicenter study aimed at developing clinical, imaging, genetic, and biochemical biomarkers for the early detection and tracking of AD progression, the Alzheimer’s Disease Neuroimaging Initiative (ADNI) provides comprehensive clinical diagnostic data that offer a holistic view of AD across multiple domains (Petersen et al., 2010). The research on AD based on time series has received attention from the academic community recently. Liang et al. proposed a multi-task learning framework that can adaptively impute missing values and predict AD progression over time from a subject’s historical measurements including MRI volumetric measurements, trajectories of a cognitive score and clinical status (Liang et al., 2021). Ho et al. proposed a bidirectional progressive recurrent network with imputation (BiPro) that uses longitudinal data to forecast clinical diagnoses and phenotypic measurements (Ho et al., 2022). El-Sappagh et al. proposed a novel two-stage deep learning AD progression detection framework based on information fusion of several patient longitudinal multivariate modalities (El-Sappagh et al., 2022). However, many prediction methods using ADNI datasets solely focus on the feature dimension of the AD data, disregarding the temporal dimension. This oversight leads to inaccurate predictions because crucial changes in the clinical features of temporal trends are missing. Moreover, during the aging process of the healthy elderly, various physical functions and conscious minds fluctuate or degrade relatively, interfering with the detection of various biomarkers (Ezzati et al., 2019). Therefore, it is challenging to predict AD progression based on time and feature dimensions while simultaneously alleviating the interference caused by natural aging to reliably differentiate between normal cognitive aging, MCI, and AD.

An effective algorithm is essential for analyzing AD clinical data. Principal component analysis (PCA) is a classical algorithm that maps the data points in high-dimensional space into lower-dimensional space to extract the main components of features and reveal their main characteristics, and is widely used in machine learning and data mining (He et al., 2021). Because of its ability to retain useful information and remove redundant information as much as possible from high-dimensional data, PCA is well-suited for AD data analysis compared to other analytical methods because AD data have high dimensionality and a small number of samples. Thus, based on PCA, a novel machine learning approach called Tensorized contrastive PCA (T-cPCA) was proposed in this study to develop an AD longitudinal clinical data representation for AD progression prediction and AD subtype identification, with the advantage of eliminating the effects of natural aging. T-cPCA can eliminate the effects of natural aging to capture low-dimensional structures enriched in the target dataset relative to the background data, providing a more accurate analysis of AD data. Based on the ADNI dataset, T-cPCA was applied to obtain the fusion features of the time and feature dimensions and was further used for AD progression prediction. Moreover, as a concept focusing on the characteristics of the typical pathological changes in AD combined with multiple groups of biomarkers (Ferreira et al., 2021), AD subtypes have a wide range of value-in-use and prospects for clinical application. To overcome the current limitations of horizontal AD subtypes and identify longitudinal AD subtypes with significant patterns, we identified four clinical AD subtypes and four representative features within each subtype.

In this context, this study proposed a novel multidimensional time series representation method termed as Tensorized contrastive Principal Component Analysis (T-cPCA) for predicting AD progression and identifying AD clinical subtypes. In AD progression prediction with stratified three-fold cross validation, T-cPCA delivers the highest ACC comparing with 6 typical PCA extension methods. Ablation experiments indicated the effectiveness of fusion features for AD progression prediction. Moreover, the identified AD clinical subtypes can be further used to improve the prediction accuracy, which incarnates that the discovery of AD clinical subtypes is a critical step toward precision medicine for this devastating disease. In addition to the effectiveness in saliency features and pathogenic circuits, the clinical manifestations and targeted treatment of the AD subtypes are discovered for AD pathophysiological mechanism research, which brings new insights for understanding the mechanisms underlying the pathogenesis of AD and paves the way for the early diagnosis.

## 2 Materials and methods

### 2.1 Overview of T-cPCA

**Figure 1:**
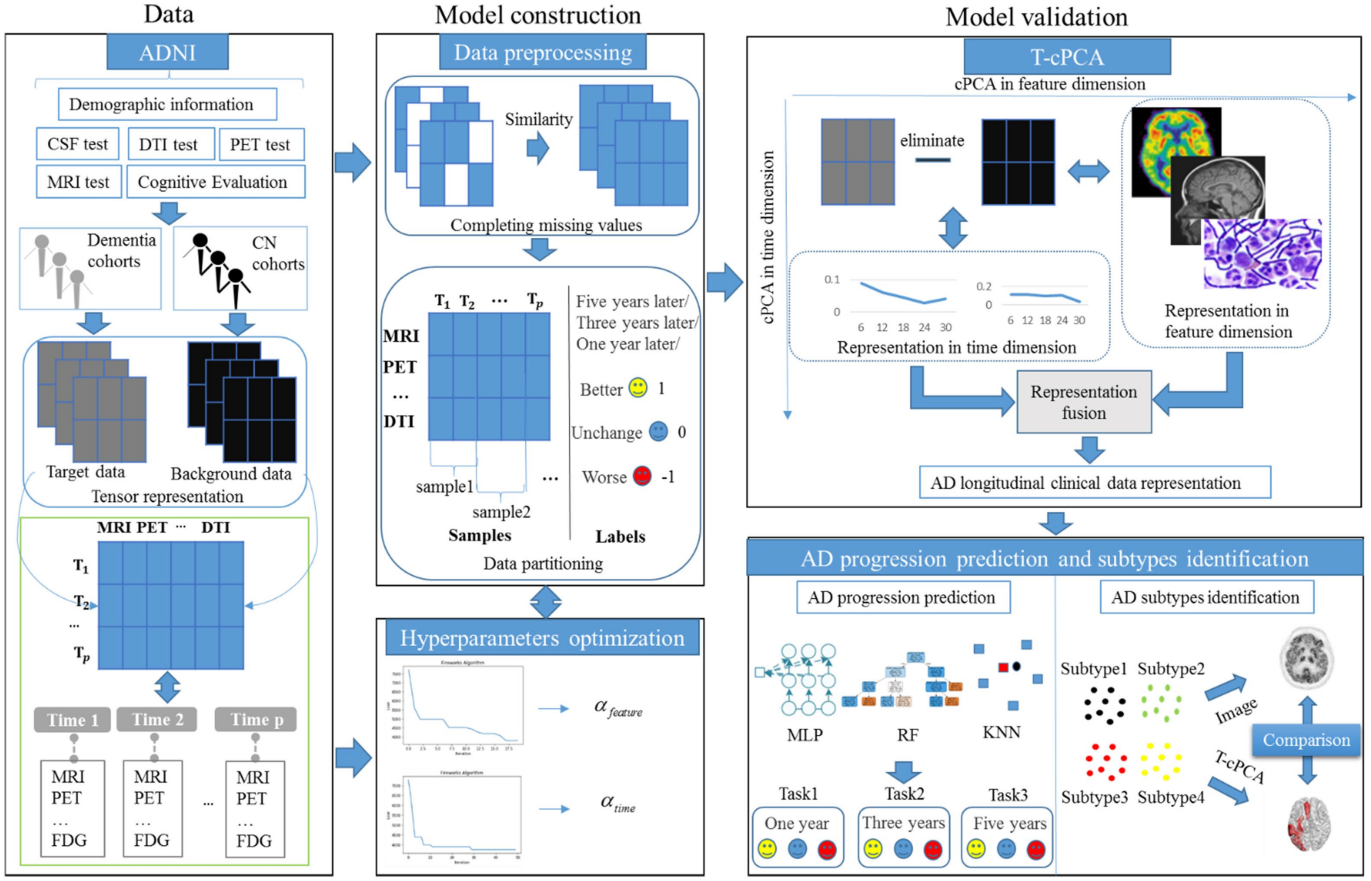
Modeling framework and overall strategy.

As an extension of contrastive PCA (cPCA), which aims to determine contrastive principal components (cPCs) that maximize the variance in the target dataset and minimize the variance in the background dataset (Yu et al., 2020; Yu and Liu, 2020), T-cPCA adopts multidimensional clinical data as tensors first. We included patients who had never suffered from AD in the cognitively normal (CN) cohort and those who had dementia in the dementia cohort. Considering the feature and time dimensions, we conducted a comparative PCA on these two dimensions to determine the cPCs on each dimension. Finally, AD clinical representation was obtained by integrating features from two dimensions for AD progression prediction and clinical subtype identification (Figure 1).

The principle of T-cPCA is as follows (Equations 1–7). Suppose we have a multidimensional time series dataset $={\left\{S_{i}\right\}}_{i=1}^{N}$ . Here $S_{i}$ can be written as tensor, the dimension of the horizontal axis represents various medical testing features (MRI, PET, etc.), and the dimension of the vertical axis represents the time span ( $T_{1}$ , $T_{2}$ ,…, $T_{P}$ ), which number of features is k , as well as span of time is p . Firstly, we partition the datasets, and then get the target data $S^{\mathrm{Tar}}={\left\{S_{i}^{\mathrm{Tar}}\right\}}_{i=1}^{N_{1}}$ and its corresponding labels, as well as background dataset $S^{\mathrm{Back}}={\left\{S_{i}^{\mathrm{Back}}\right\}}_{i=1}^{N_{2}}$ . Considering the feature dimension and time dimension of data, we need to conduct comparative principal component analysis on these two dimensions, respectively, in order to find the cPCs on each dimensions.

(1) $$C_{\mathrm{Tar}}^{\mathrm{feature}}=\frac{1}{N_{1}}\overset{N_{1}}{\underset{i=1}{\sum }}{\left(S_{i}^{\mathrm{Tar}}-\bar{S_{i}^{\mathrm{Tar}}}\right)}^{T}\left(S_{i}^{\mathrm{Tar}}-\bar{S_{i}^{\mathrm{Tar}}}\right)$$

(2) $$C_{\mathrm{Back}}^{\mathrm{feature}}=\frac{1}{N_{2}}\overset{N_{2}}{\underset{i=1}{\sum }}{\left(S_{i}^{\mathrm{Back}}-\bar{S_{i}^{\mathrm{Back}}}\right)}^{T}\left(S_{i}^{\mathrm{Back}}-\bar{S_{i}^{\mathrm{Back}}}\right)$$

We aim to find:

(3) $$\underset{v\in R_{\mathrm{unit}}^{d}}{\mathrm{argmax}}v^{T}\left(C_{\mathrm{Tar}}^{\mathrm{feature}}-\alpha_{\mathrm{feature}}C_{\mathrm{Back}}^{\mathrm{feature}}\right)v$$

And then we get matrix $M^{\mathrm{feature}}$ , which is composed of eigenvectors corresponding to the top $m_{\mathrm{feature}}$ cPCs, where $m_{\mathrm{feature}}\leq k$ .

(4) $$C_{\mathrm{Tar}}^{\mathrm{time}}=\frac{1}{N_{1}}\overset{N_{1}}{\underset{i=1}{\sum }}\left(S_{i}^{\mathrm{Tar}}-\bar{S_{i}^{\mathrm{Tar}}}\right){\left(S_{i}^{\mathrm{Tar}}-\bar{S_{i}^{\mathrm{Tar}}}\right)}^{T}$$

(5) $$C_{\mathrm{Back}}^{\mathrm{time}}=\frac{1}{N_{2}}\overset{N_{2}}{\underset{i=1}{\sum }}\left(S_{i}^{\mathrm{Back}}-\bar{S_{i}^{\mathrm{Back}}}\right){\left(S_{i}^{\mathrm{Back}}-\bar{S_{i}^{\mathrm{Back}}}\right)}^{T}$$

We aim to find:

(6) $$\underset{v\in R_{\mathrm{unit}}^{d}}{\mathrm{argmax}}v^{T}\left(C_{\mathrm{Tar}}^{\mathrm{time}}-\alpha_{\mathrm{time}}C_{\mathrm{Back}}^{\mathrm{time}}\right)v$$

And then we get matrix $M^{\mathrm{time}}$ , which is composed of eigenvectors corresponding to the top $m_{\mathrm{time}}$ cPCs, where $m_{\mathrm{time}}\leq p$ .

The final mapping data can be converted into:

(7) $$S_{i}^{\mathrm{change}}={M^{\mathrm{time}}}^{T}S_{i}M^{\mathrm{feature}}$$

The details of T-cPCA is provided in Supplement material A.

### 2.2 Participants and measurements

The ADNI database was initiated in 2003 by several national institutes, including the Food and Drug Administration, private pharmaceutical companies, and nonprofit organizations in the United States. This longitudinal multicenter study aimed to develop clinical, imaging, genetic, and biochemical biomarkers for the early detection and tracking of AD. The ADNI dataset is updated regularly, and the longitudinal clinical data of 1,631 participants had been collected until 2022. The TADPOLE challenge provides a standardized dataset exported by the ADNI with the richest clinical features for AD research. In the experiments, we utilized the dataset provided by TADPOLE, which includes the following: (1) amyloid and CSF biomarkers of tau deposition; (2) various biomarker analysis methods, positron emission tomography with several different tracers: fluorodeoxyglucose (FDG), AV45 (amyloid), AV1451 (Tau proteins), and DTI; (3) cognitive evaluation performed in the presence of clinical experts; (4) genetic information extracted from DNA, such as APOE4 expression level; (5) demographic information, including age, gender, education level, race, and marital status. In this study, the dataset provided by the TADPOLE challenge included the clinical diagnostic data of 1,631 participants which are divided into CN, MCI, and AD (Table 1).

**Table 1:** Baseline characteristics of participants in TADPOLE.

		CN	MCI	AD
Number of individuals		417	872	342
Age at baseline(years)	≤70	72	299	80
	70<age≤80	270	400	168
	>80	75	173	94
Gender	% Male	50%	59%	55%
Race	% White	93%	93%	93%
Marital status at baseline	% Married	68%	77%	84%
MMSE at baseline		29.0 ± 6.1	27.4 ± 8.4	23.1 ± 4.0

The longitudinal nature of the ADNI dataset was evident in its continuous collection of clinical diagnostic data for each participant unless they passed away or withdrew voluntarily for other reasons. These long-term data provide a robust foundation for further research on AD.

### 2.3 Model parameter selection

In T-cPCA, increasing the values of hyperparameters $\alpha_{\mathrm{feature}}$ and $\alpha_{\mathrm{time}}$ , which denote the trade-off between the dementia and CN cohorts, can improve the elimination of natural aging effects. Considering the complexity of the T-cPCA algorithm, we developed an intelligent evolutionary algorithm to rapidly and effectively determine the optimal solution for the hyperparameters. With the advantages of strong global and local searching capabilities, the firework algorithm is a swarm intelligence algorithm widely used for solving optimization problems in various domains, including image recognition and spam detection (Zheng et al., 2015). The information is exchanged among fireworks and is characterized by the number of explosion sparks and explosion radius, improving the suitability of the firework algorithm for the high-dimensional small-sample characteristics of AD data. Therefore, we propose an improved extension of the traditional firework algorithm specifically tailored for AD data to enhance its computational efficiency. For details of the algorithm, please refer to Supplementary material A.

## 3 Results

### 3.1 AD progression prediction

Following the principle of preserving comprehensive information from feature and time dimensions, we initially aligned the data in the time dimension. Subsequently, missing values in the diagnostic data were supplemented using similar diagnostic results. We used the data of patients who had never suffered from AD as background data, the data of patients who had suffered from AD as target data, and the change in future disease development as the target data label, denoted better (1), worse (−1), and unchanged (0). The prediction time spans were selected as 1, 3, and 5 years, respectively (Table 2).

**Table 2:** Baseline characteristics of samples in experiments.

	Target data	Background data
**1-year**		
Number of samples	1909	884
Time dimension	8	8
Feature dimension	872	872
Label proportion	67:198:1644(1:-1:0)	–
**3-year**		
Number of samples	1,235	506
Time dimension	8	8
Feature dimension	872	872
Label proportion	71:232:932(1:-1:0)	-
**5-year**		
Number of samples	534	196
Time dimension	8	8
Feature dimension	872	872
Label proportion	41:151:342(1:-1:0)	–

The comparative experiments were implemented among T-cPCA and original multidimensional time series (OR), and the calculated statistical features from the time series including mean value, standard deviation, maximum, minimum, variance, skewness and kurtosis (SOR), and six typical PCA representation methods, comprising the two-dimensional PCA (2DPCA), kernal PCA with radial basis function kernel (PCA1), kernal PCA with rational quadratic kernel (PCA2), kernal PCA with linear kernel (PCA3), kernal PCA with polynomial kernel (PCA4), and kernal PCA with sigmoid kernel (PCA5), and two ablation models (T-cPCA only with feature representation and T-cPCA only with time representation) with three popular supervised machine-learning algorithms (Multilayer Perceptron, Random Forest, and K-Nearest Neighbors algorithm) to verify the effectiveness of the proposed method. The technical details about these machine learning algorithms are shown in A.5 section in the Supplementary materials. The prediction tasks were 1-, 3-, and 5-year AD progression predictions with a stratified three-fold cross-validation. The evaluation indices of the models were the accuracy, recall value, and F1 score.

In contrast to the six typical PCA representation methods, the representation obtained by T-cPCA had the highest classification accuracy and delivered the highest specificity for progression prediction using three popular supervised machine-learning algorithms (Table 3). And the prediction results in Table 3 underwent paired sample t-tests to demonstrate the significance of T-cPCA. We proposed the Null Hypothesis as $H_{0}:x_{d}\leq 0$ (The average difference between prediction performance of T-cPCA and the comparison models in three evaluation indices ≤0 ), while the Alternative Hypothesis is $H_{1}:x_{d}>0$ (The average difference between prediction performance of T-cPCA and the comparison models in three evaluation indices >0 ), and our significance level is α=0.01 . As a result. we compute theT-statistic value and obtain the *p*-value. If *p*-value ≤0.01 and T-statistic value >2 , we reject $H_{0}$ and accept $H_{1}$ , which means that the prediction performance of T-cPCA is significantly superior to that of comparison models.

**Table 3:** Performance of predictive models in the measurement of accuracy (ACC), recall value (recall), and F1 score (F1).

	OR	SOR	2DPCA	PCA1	PCA2	PCA3	PCA4	PCA5	TcPCA0	TcPCA1	TcPCA
**1-year**									**ACC**		
MLP	0.5621^†^	0.5867^†^	0.6264^†^	0.6389^†^	0.6339^†^	0.6201^†^	0.6427^†^	0.5396^†^	0.6389	0.6654	**0.6716** ^◊^
RF	0.5734^†^	0.5981^†^	0.6327^†^	0.5748^†^	0.6377^†^	0.6805^†^	0.6830	0.5924^†^	0.6742	0.6817	**0.6993** ^◊^
KNN	0.5679^†^	0.5263^†^	0.6566	0.6666^†^	0.5849^†^	0.6704	0.6679	0.6276^†^	0.6628	0.6679	**0.6729** ^◊^
**3-year**											
MLP	0.7062^†^	0.5791^†^	0.7469^†^	0.6534^†^	0.5665^†^	0.7216^†^	0.7040^†^	0.5104^†^	0.7117	0.7513	**0.7579** ^◊^
RF	0.6270^†^	0.6813^†^	0.7106^†^	0.5973^†^	0.6666^†^	0.7139^†^	0.7062	0.5885^†^	0.6963	0.7150	**0.7447** ^◊^
KNN	0.6567^†^	0.5862^†^	0.7315^†^	0.6677^†^	0.6765^†^	0.7282	0.7260	0.4950^†^	0.7326	0.7293	**0.7425** ^◊^
**5-year**											
MLP	0.7239^†^	0.6785^†^	0.7490^†^	0.4868^†^	0.5037^†^	0.6816^†^	0.6910^†^	0.4719^†^	0.7284	0.7453	**0.7659** ^◊^
RF	0.5885^†^	0.6279	0.6372^†^	0.6391^†^	0.6372	0.6466^†^	0.6429	0.6372^†^	0.6429	0.6428	**0.6485** ^◊^
KNN	0.6119^†^	0.6542^†^	0.6797^†^	0.6404^†^	0.6404^†^	0.6647	0.6685	0.5861^†^	0.6891	0.6872	**0.6891** ^◊^
**1-year**									**Recall**		
MLP	0.4585^†^	0.4515	0.4367^†^	0.3550^†^	0.3792^†^	0.4300^†^	0.4475^†^	0.3205^†^	0.4534	**0.4709**	0.4634^◊^
RF	0.4231^†^	**0.4654**	0.3900^†^	0.3255^†^	0.3906^†^	0.4249^†^	0.4286	0.3431^†^	0.4228	0.4427	0.4395^◊^
KNN	**0.4615**	0.3888^†^	0.4118	0.3333^†^	0.3648^†^	0.4123^†^	0.4127	0.3440^†^	0.4143	0.4260	0.4226^◊^
**3-year**											
MLP	**0.6227**	0.5161^†^	0.6077	0.4128^†^	0.4293^†^	0.5501^†^	0.5201^†^	0.3256^†^	0.5457	0.5871	0.5936^◊^
RF	0.4665^†^	0.5019	0.4569^†^	0.3330^†^	0.3333^†^	0.4922^†^	0.4722	0.3442^†^	0.4792	0.4839	**0.5117** ^◊^
KNN	0.5485	0.4656^†^	0.5288^†^	0.3347^†^	0.3552^†^	0.5284^†^	0.5163^†^	0.3180^†^	0.5429	0.5317	**0.5553** ^◊^
**5-year**											
MLP	0.5485	0.5492^†^	0.5710	0.3594^†^	0.3751^†^	0.5091^†^	0.5039	0.2969^†^	0.5459	0.5354	**0.5669** ^◊^
RF	**0.4282**	0.4133	0.3323^†^	0.3333^†^	0.3350^†^	0.3415	0.3381	0.3323^†^	0.3403	0.3405	0.3510^◊^
KNN	0.4880^†^	0.4607^†^	0.4977^†^	0.3833^†^	0.3333^†^	0.4440^†^	0.4498^†^	0.3203^†^	0.5129	0.4953	**0.5129** ^◊^
**1-year**									**F1**		
MLP	0.4554^†^	0.4492	0.4313^†^	0.3410^†^	0.3734^†^	0.4361^†^	0.4548^†^	0.3244^†^	0.4520	**0.4606**	0.4597^◊^
RF	0.3910^†^	**0.4574**	0.3760^†^	0.3127^†^	0.3848^†^	0.4209^†^	0.4195	0.3290^†^	0.4086	0.4361	0.4342^◊^
KNN	**0.4602**	0.3701^†^	0.4139	0.2666^†^	0.3032^†^	0.4129^†^	0.4136	0.3189^†^	0.4177	0.4313	0.4255^◊^
**3-year**											
MLP	**0.6198**	0.5042^†^	0.6073	0.4124^†^	0.3816^†^	0.5574^†^	0.5387^†^	0.3266^†^	0.5575	0.5963	0.6096^◊^
RF	0.4381^†^	0.5055	0.4547^†^	0.3150^†^	0.2666^†^	0.5032^†^	0.4819	0.3347^†^	0.4837	0.4774	**0.5132** ^◊^
KNN	**0.5561**	0.4606^†^	0.5108	0.2695^†^	0.3120^†^	0.5139^†^	0.5046^†^	0.3081^†^	0.5254	0.5148	0.5441^◊^
**5-year**											
MLP	**0.6134**	0.5521	0.5771^†^	0.3169^†^	0.3310^†^	0.5042^†^	0.5087	0.2929^†^	0.5415	0.5417	0.5605^◊^
RF	0.4050	**0.5141**	0.2594^†^	0.2598^†^	0.2679	0.2768^†^	0.2699^†^	0.2594^†^	0.2772	0.3428	0.2997^◊^
KNN	0.4905^†^	0.4579^†^	0.5092^†^	0.2602^†^	0.2602^†^	0.4479^†^	0.4689	0.2843^†^	0.5293	0.5094	**0.5293** ^◊^

^†^Means that the prediction performance of the comparison models is significantly worse than that of T-cPCA (excluding ablation models). ^◊^means that the prediction performance of T-cPCA is significantly better than that of more than half of the comparison models (excluding ablation models). The bold values are the best results in each row.

Table 4 is the standard deviations of predictions Table 3, which are obtained from ten runs of all models.

**Table 4:** The standard deviations of the model prediction performance in Table 3.

	OR	SOR	2DPCA	PCA1	PCA2	PCA3	PCA4	PCA5	TcPCA0	TcPCA1	TcPCA
**1-year**									**ACC**		
MLP	0.0338	0.0359	0.0323	0.0562	0.0718	0.0312	0.0287	0.0285	0.0243	0.0323	0.0272
RF	0.0340	0.0364	0.0339	0.0305	0.0275	0.0300	0.0303	0.0288	0.0375	0.0327	0.0391
KNN	0.0237	0.0336	0.0337	0.0330	0.0334	0.0282	0.0341	0.0314	0.0307	0.0325	0.0345
**3-year**											
MLP	0.0273	0.0275	0.0348	0.0609	0.1066	0.0282	0.0360	0.0283	0.0264	0.0317	0.0278
RF	0.0370	0.0435	0.0325	0.0274	0.0415	0.0372	0.0448	0.0352	0.0380	0.0357	0.0318
KNN	0.0351	0.0233	0.0309	0.0291	0.1048	0.0338	0.0355	0.0308	0.0376	0.0282	0.0371
**5-year**											
MLP	0.0376	0.0323	0.0321	0.0663	0.1017	0.0374	0.0232	0.0260	0.0334	0.0237	0.0315
RF	0.0345	0.0418	0.0345	0.0331	0.0325	0.0367	0.0332	0.0320	0.0352	0.0415	0.0340
KNN	0.0315	0.0414	0.0281	0.0306	0.0371	0.0324	0.0333	0.0302	0.0320	0.0327	0.0320
**1-year**									**Recall**		
MLP	0.0352	0.0353	0.0365	0.0220	0.0191	0.0378	0.0286	0.0296	0.0295	0.0337	0.0294
RF	0.0266	0.0236	0.0196	0.0098	0.0132	0.0182	0.0202	0.0086	0.0232	0.0239	0.0268
KNN	0.0221	0.0256	0.0231	0.0020	0.0168	0.0176	0.0215	0.0212	0.0183	0.0227	0.0219
**3-year**											
MLP	0.0367	0.0361	0.0453	0.0266	0.0361	0.0306	0.0403	0.0200	0.0418	0.0328	0.0467
RF	0.0254	0.0278	0.0202	0.0110	0.0243	0.0235	0.0264	0.0163	0.0239	0.0283	0.0214
KNN	0.0379	0.0266	0.0317	0.0150	0.0364	0.0299	0.0261	0.0221	0.0316	0.0294	0.0310
**5-year**											
MLP	0.0443	0.0344	0.0408	0.0270	0.0252	0.0499	0.0358	0.0210	0.0449	0.0388	0.0424
RF	0.0220	0.0308	0.0102	0.0020	0.0131	0.0124	0.0108	0.0053	0.0191	0.0272	0.0086
KNN	0.0283	0.0370	0.0279	0.1102	0.0183	0.0303	0.0354	0.0162	0.0352	0.0302	0.0352
**1-year**									**F1**		
MLP	0.0374	0.0383	0.0355	0.0309	0.0323	0.0346	0.0308	0.0297	0.0289	0.0354	0.0311
RF	0.0340	0.0258	0.0327	0.0248	0.0261	0.0310	0.0364	0.0261	0.0372	0.0328	0.0441
KNN	0.0241	0.0357	0.0286	0.0105	0.0328	0.0234	0.0257	0.0256	0.0215	0.0275	0.0258
**3-year**											
MLP	0.0293	0.0381	0.0455	0.0412	0.0535	0.0325	0.0411	0.0266	0.0378	0.0303	0.0439
RF	0.0249	0.0284	0.0317	0.0263	0.0383	0.0347	0.0366	0.0307	0.0315	0.0286	0.0279
KNN	0.0391	0.0325	0.0347	0.0309	0.0766	0.0387	0.0286	0.0306	0.0343	0.0328	0.0362
**5-year**											
MLP	0.0453	0.0396	0.0451	0.0328	0.0455	0.0455	0.0359	0.0219	0.0448	0.0389	0.0426
RF	0.0321	0.0407	0.0238	0.0106	0.0267	0.0282	0.0240	0.0151	0.0379	0.0475	0.0238
KNN	0.0322	0.0402	0.0322	0.0075	0.0196	0.0363	0.0427	0.0248	0.0425	0.0345	0.0425

With regard to the prediction results of different time spans, the AD clinical representation by T-cPCA showed the highest accuracy in the tasks of 1-, 3-, and 5-year prediction, suggesting that T-cPCA could capture the long-term change characteristics of AD development. Moreover, MLP is more effective in predicting AD progression based on T-cPCA. We infer that T-cPCA, as an extension of PCA series algorithms, can extract effective features that integrate the fusion information of the time and feature dimensions, thus providing novel insights for analyzing the internal trend of AD data.

To further demonstrate the effectiveness of T-cPCA, four typical deep neural network structures including convolutional neural network (CNN), Long Short Term Memory network (LSTM), gated recurrent neural network (GRU) and bidirectional LSTM (BiLSTM) are applied for AD progression prediction based on T-cPCA and original multidimensional time series (Table 5). The deep neural network for comparison is constructed as two layer typical network structure and four layer fully connected network structure. The experimental results indicate that MLP based on T-cPCA delivers the highest ACC among the other prediction models especially deep learning models, which means that deep learning is not as effective as MLP in predicting AD progression.

**Table 5:** Performance of MLP and four typical deep neural networks based on T-cPCA and original multidimensional time series in the measurement of accuracy (ACC), recall value (recall), and F1 score (F1).

	1-year			3-year			5-year		
	ACC	Recall	F1	ACC	Recall	F1	ACC	Recall	F1
OR + CNN	0.4877	**0.5091**	**0.4855**	0.5355	0.5615	0.5308	0.6503	0.5812	0.6022
TcPCA + CNN	0.4307	0.4321	0.4265	0.5326	0.5267	0.5135	0.5098	0.4974	0.4917
OR + LSTM	0.4723	0.5071	0.4724	0.5967	0.5717	0.5740	0.5681	0.5708	0.5623
TcPCA + LSTM	0.4801	0.4765	0.4757	0.6272	0.6166	0.6167	0.4958	0.5158	0.4969
OR + GRU	0.4693	0.4795	0.4644	0.6380	0.6062	0.6008	0.6647	0.5768	0.5725
TcPCA+ GRU	0.4621	0.4649	0.4593	0.6371	0.6053	0.6137	0.5514	0.5805	0.5468
OR + BiLSTM	0.5027	0.5025	0.4620	0.6882	0.6327	**0.6509**	0.5317	0.5756	0.5437
TcPCA + BiLSTM	0.4617	0.4599	0.4572	0.6584	**0.6446**	0.6404	0.6219	0.5770	0.5880
OR + MLP	0.5621	0.4585	0.4554	0.7062	0.6227	0.6198	0.7239	**0.6085**	**0.6134**
TcPCA + MLP	**0.6716**	0.4634	0.4597	**0.7579**	0.5936	0.6096	**0.7659**	0.5669	0.5605

The bold values are the best results in each column.

### 3.2 AD clinical subtypes identification

Many genetic, metabolic, and clinical studies have provided evidence for the existence of distinct AD subtypes. The identification of these subtypes helps improve AD biomarker identification, targeted pathological research, correct patient diagnosis, and efficient drug development. In this section, a clustering algorithm called hierarchical clustering is applied to the clinical representation obtained by T-cPCA to identify AD clinical subtypes. By maximizing the clustering effectiveness evaluation index called the silhouette score, we identified four clinical AD subtypes. For each subtype, three machine-learning algorithms with the same hyperparameters were used to verify the effectiveness of clustering results. The details are shown in Supplementary material B.

After clustering, compared with the prediction results before clustering, the classification results obtained by applying classifiers with the same parameters in each cluster showed considerable improvement, particularly for the key evaluation index (ACC), among which the one-year prediction task showed an almost 10% improvement in the ACC (Table 6). On the one hand, it demonstrated the effectiveness of the four identified AD clinical subtypes. On the other hand, the AD progression prediction performance can be further improved by training classifiers in different clinical subtypes. For the five-year prediction task with the KNN classifier, the prediction performance of the model decreased after clustering, owing to the small amount of data. Overall, the experimental results show that the clinical representation extracted by T-cPCA is significant and can provide a solid foundation for future research.

**Table 6:** Comparison of performance in task of AD progression prediction before and after clustering.

Classifier	Before cluster (best)			After cluster			ACC	Recall	F1	ACC	Recall	F1
**1-year**						
MLP	0.6716	**0.4634**	**0.4597**	**0.8197**	0.4518	0.4502
RF	0.6993	**0.4395**	**0.4342**	**0.8501**	0.4043	0.4091
KNN	0.6729	0.4226	0.4255	**0.8417**	**0.4323**	**0.4308**
**3-year**						
MLP	0.7579	**0.5936**	**0.6096**	**0.7797**	0.5717	0.5721
RF	0.7447	**0.5117**	**0.5132**	**0.7676**	0.4723	0.4663
KNN	0.7425	**0.5553**	**0.5441**	**0.7920**	0.5139	0.5170
**5-year**						
MLP	0.7659	0.5669	0.5605	**0.7856**	**0.6439**	**0.6519**
RF	0.6485	0.351	0.2997	**0.6639**	**0.4196**	**0.4016**
KNN	**0.6891**	**0.5129**	**0.5293**	0.6671	0.4514	0.4312

The bold values are the best results in the comparison between Before cluster (best) and After cluster.

## 4 Discussion

### 4.1 Horizontal characterization of AD clinical subtypes

The clinical features of the different AD subtypes may hold promise for the early diagnosis of AD. Therefore, the Gini index was used to calculate the importance of the features in each subtype to identify the key clinical features of the four AD clinical subtypes. The gini index is a kind of statistical indicator to measure feature importance in the Random Forest (RF) algorithm, and it has been widely used in many fields. Suppose we use $VIM_{j}^{\mathrm{gini}}$ to denote the $j^{th}$ feature’s Gini index, which demonstrates the average change of node splitting impurity of the $j^{th}$ feature in all RF decision trees. GI denotes the Gini index. Assuming that there are n features $x_{1},x_{2},\ldots ,x_{n}$ , The Gini index of node q of the ith tree is calculated as (Equations 8–11):

(8) $$GI_{q}^{\left(i\right)}=\overset{\left|C\right|}{\underset{c=1}{\sum }}\underset{c^{\prime }\neq c}{\sum }p_{qc}^{\left(i\right)}p_{qc^{\prime }}^{\left(i\right)}=1-\overset{\left|C\right|}{\underset{c=1}{\sum }}{\left(p_{qc}^{\left(i\right)}\right)}^{2}$$

Here C indicates that there are C categories, $p_{qc}$ denotes the proportion of category c in node q . The importance of $x_{j}$ in node q of the $i^{th}$ tree is the change of Gini index before and after branching. The calculation process is as following:

(9) $$VIM_{jq}^{\mathrm{gini}\left(i\right)}=GI_{q}^{\left(i\right)}-GI_{l}^{\left(i\right)}-GI_{r}^{\left(i\right)}$$

Among them, $GI_{l}$ and $GI_{r}$ respectively denote the Gini index of two new nodes after branching. If the variable $x_{j}$ appears m times in the $i^{th}$ tree, then the importance of feature $x_{j}$ in the $i^{th}$ tree is:

(10) $$VIM_{j}^{\mathrm{gini}\left(i\right)}=\overset{m}{\underset{n=1}{\sum }}VIM_{jn}^{\mathrm{gini}\left(i\right)}$$

Above all, the Gini importance of the $j^{th}$ feature in RF is defined as:

(11) $$VIM_{j}^{\mathrm{gini}}=\overset{N}{\underset{i=1}{\sum }}VIM_{j}^{\mathrm{gini}\left(i\right)}$$

Among them, N is the number of trees.

Finally, we normalize all Gini importance scores.

(12) $$VIM_{j}^{\mathrm{gini}}=\frac{VIM_{j}^{\mathrm{gini}}}{\sum_{i=1}^{n}VIM_{i}^{\mathrm{gini}}}$$

**Figure 2:**
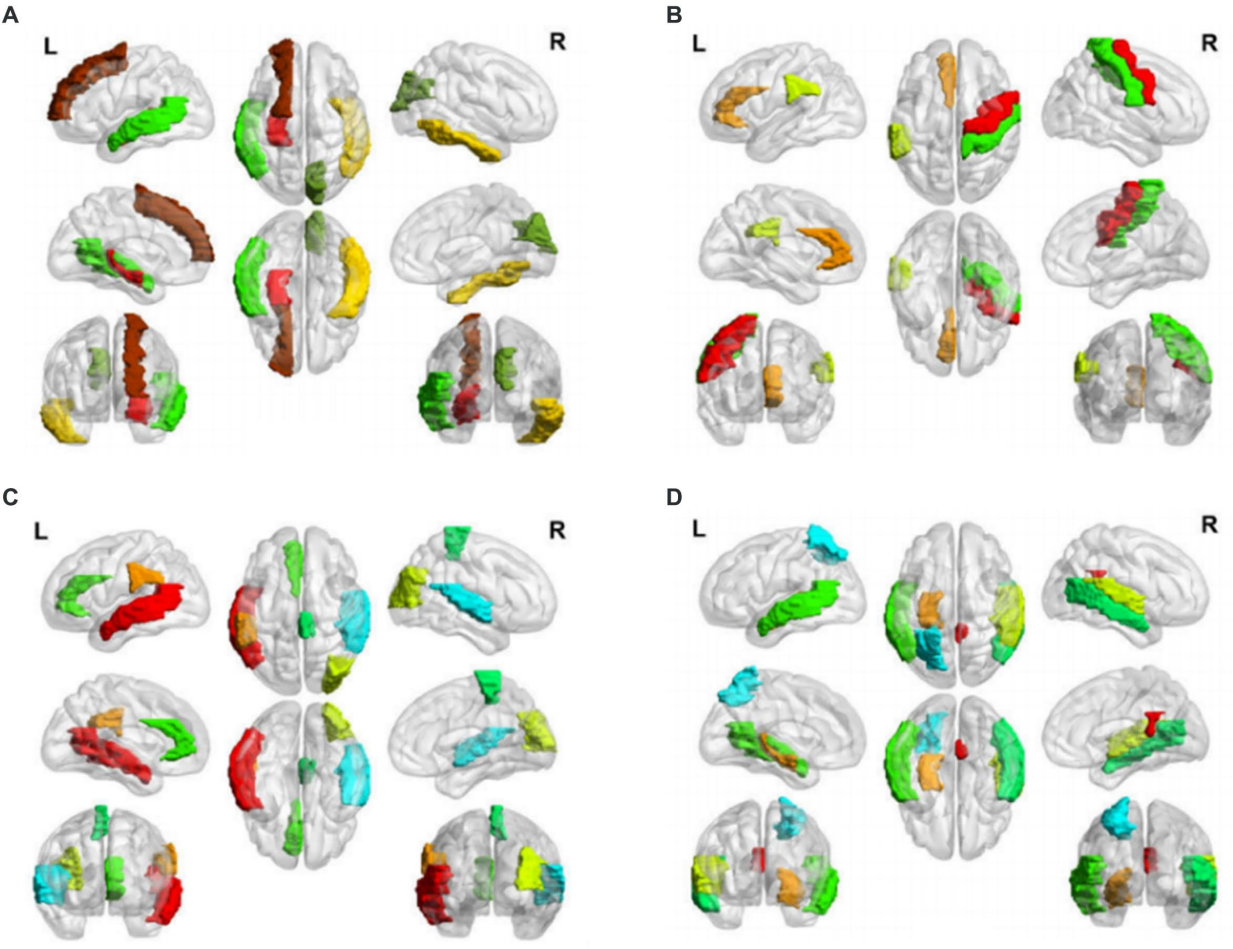
Salient-affected brain regions for four clinical subtypes. Salient-affected brain regions in **(A)** first, **(B)** second, **(C)** third, and **(D)** fourth clinical AD subtypes. For each figure, the first row from left to right are the lateral views of the left hemisphere, topside, and lateral view of the right hemisphere. The second row from left to right are medial views of the left hemisphere, bottom side, and medial view of the right hemisphere. The third row shows the frontal side and backside.

We selected features with a Gini index higher than 0.02 as the representative features of the different subtypes. The salient brain regions affected by the four clinical AD subtypes are shown in Figure 2. The details of the top 10 important features of four subtypes are provided in Supplementary material B.

In the first clinical AD subtype, the salient-affected brain regions were located in the corpus callosum, right cuneus, left inferior temporal gyrus, left superior frontal gyrus, left transverse temporal gyrus, left middle temporal gyrus, left superior frontal gyrus, and left hippocampus (Figure 2A). Alterations in the corpus callosum emerged as an early manifestation of this AD subtype progression. As the largest white matter structure, the corpus callosum receives blood from several major arterial systems and plays a critical role in the onset of AD (Das et al., 2021). Accumulation of amyloid-beta peptide ( Αβ ) is related to callosal myelination, leading to an imbalance in glial cells, an increased presence of phagocytic microglia and reactive astrocytes, and reduced numbers of oligodendrocyte progenitor cells (Aires et al., 2022). The mean diffusivity of the corpus callosum measured using DTI showed a significant decrease in fractional anisotropy among patients with AD (Xiao et al., 2022). MRI has revealed that atrophy of the posterior corpus callosum is positively associated with apathy in patients with AD (Yu et al., 2020). Additionally, the compromised white matter microstructure in the posterior section of the corpus callosum is associated with poorer semantic fluency (Sánchez et al., 2020). Furthermore, combination therapy by Donepezil and Rivastigmine has demonstrated significant improvements in the size of the corpus callosum in patients with severe Alzheimer’s disease (Khasawneh et al., 2022).

In the second clinical AD subtype, prominent changes were observed in specific brain regions, including the left anterior cingulate gyrus, left supramarginal gyrus, right precentral gyrus, and right precentral gyrus (Figure 2B). The affected areas primarily involve the cingulate gyrus and supramarginal gyrus, which are crucial hubs for information processing and regulation within the brain (Yuan et al., 2022). Research has demonstrated that a higher tau signal (CSF Αβ 42/40 ratio) and reduced gray matter density in the posterior cingulate cortex and angular gyrus are associated with decreased parietal functional connectivity in individual patients (Berron et al., 2020), leading to memory decline (Yasar et al., 2020). Blood oxygenation level-dependent signals measured using resting-state functional magnetic resonance imaging have been associated with AD development (Zheng et al., 2020). In patients with AD, cerebral blood flow (CBF) decreases from the early stages as processes preceding and following the onset of cerebrovascular risk factors, or stroke may trigger amyloid-beta deposition in the precuneus/posterior cingulate cortex. The epsilon 4 allele of the apolipoprotein E (APOE) gene may accelerate age-related cortical thickening and reduction in CBF in the anterior cingulate cortex (Hays et al., 2020). In addition, the posterior cingulate gyrus is particularly activated during the recollection of personal events and inference of others’ mental states, and dysfunction in this area contributes to cognitive decline in tasks involving verbal information storage, drawing abilities, and nonverbal abstract reasoning among individuals with AD (Takenoshita et al., 2020). Significant correlations have been observed between the functional connectivity of the anterior cingulate cortex and episodic memory dysfunction and executive function impairments. Evidence suggests that angiotensin II type-1 receptor blockers may protect against memory decline by reducing the rates of amyloid-beta accumulation in this AD subtype (Ouk et al., 2021).

For the third clinical AD subtype, the salient-affected brain regions were located in left temporal lobe, left supramarginal gyrus, right occipital lobe, right superior temporal gyrus, left anterior cingulate gyrus, and right paracentral lobule (Figure 2C). The affected brain regions were primarily located in the temporal lobe. Seizures that occur early in the course of AD are likely to originate from the mesial temporal lobe, which is one of the first structures affected by Alzheimer’s pathology and one of the most epileptogenic regions in the brain. Genetic mutations associated with AD increase the tau levels, and the accumulation of tau linearly increases neuronal hyperexcitability, leading to seizures (Zawar and Kapur, 2023). The presence of baseline CSF Ptau is related to the loss of structural stability in connectivity within the medial temporal lobe (Chen et al., 2020). During the early stages of MCI, hyperconnectivity within the ventral medial temporal lobe structures and hypoconnectivity between the dorsal medial temporal lobe regions and the anterior/posterior midline default-mode network nodes are crucial biomarkers for early AD diagnosis, which can further progress to cortical atrophy in the occipital temporal lobe (Sintini et al., 2020). The clinical symptoms include temporal lobe epilepsy, situational amnesia, and worse executive functioning, language, and attention (Visser et al., 2020). The entrainment of neural oscillations in the occipital cortices through external rhythmic visual stimuli shows promise as a novel therapy for AD patients with this subtype (Wiesman et al., 2021). Rapamycin, an immune system inhibitor and a longevity drug, may be a potential treatment for this AD subtype by rescuing proteins in the temporal lobe (Wang et al., 2019).

For the fourth clinical AD subtype, the salient-affected brain regions were located in the left entorhinal cortex, left middle temporal gyrus, right cingulate gyrus, right transverse temporal gyrus, right bankssts, left parietal lobe, and right middle temporal gyrus (Figure 2D). As one of the earliest sites showing pathological changes, the entorhinal cortex plays a critical role in the development of this AD subtype. Aging of the entorhinal cortex is associated with increased expression levels of APP genes and MAPT genes, resulting in significant accumulation of β-amyloid (Aβ) and neurofibrillary tangles during the amnestic MCI phase of AD (Li et al., 2022). Additionally, a decrease in CBF in the entorhinal cortex precedes tau deposition and contributes to memory impairment and spatial navigation deficit, leading to disorientation and wandering behavior. Neuronal loss is considered the primary manifestation of AD development, accompanied by a decrease in microglia and proliferation of astrogliosis in this subtype (Astillero-Lopez et al., 2022). The grid-cell network of the entorhinal cortex, which is considered one of the earliest neurodegenerative regions, is crucial for path integration in humans and rodents (Segen et al., 2022). The anterolateral entorhinal cortex plays a significant role in memory retention, and differences in its volume are associated with the performance on neuropsychological tests for AD (Yeung et al., 2021). Deep brain stimulation has shown promise in improving the cognitive function and has prompted clinical trials for the early treatment of AD (Yu et al., 2019). Chemical-protein interaction analysis has revealed that valproic acid is a potential therapeutic agent that can prevent AD progression in this subtype (Bottero et al., 2021).

We have demonstrated that the unique characteristics of the four AD clinical subtypes can effectively reveal multiple mechanisms and heterogeneous clinical manifestations (Table 7). This explains why AD is a syndrome with multiple coexisting mechanisms, providing a favorable basis for further research on multitarget drug interventions in clinical practice. On the one hand, our data validated the effectiveness of the proposed method. On the other hand, our method provides an important basis for early diagnosis and appropriate treatment of AD.

**Table 7:** Difference among four clinical subtypes.

	Affected brain regions	Clinical manifestations	Treatment
Subtype 1	corpus callosum, right cuneus, left inferior temporal gyrus, left superior frontal gyrus, left transverse temporal gyrus, left middle temporal gyrus, left superior frontal gyrus and left hippocampus	poorer semantic fluency, apathy	Donepezil and rivastigmine
Subtype 2	left anterior cingulate gyrus, left supramarginal gyrus, right precentral gyrus and right precentral gyrus	episodic memory dysfunction, executive function impairments, storage of verbal information, drawing abilities, and non-verbal abstract reasoning	Angiotensin II type 1
Subtype 3	left temporal lobe, left supramarginal gyrus, right occipital lobe, right superior temporal gyrus, left anterior cingulate gyrus and right paracentral lobule	temporal lobe epilepsy, situational amnesia, and worse executive functioning, language and attention	External rhythmic visual stimuli, rapamycin
Subtype 4	left entorhinal cortex, left middle temporal gyrus, right cingulate gyrus, right transverse temporal, right bankssts, left parietal lobe and right middle temporal gyrus	memory impairments, spatial navigation deficits, disorientation, wandering behavior	Deep brain stimulation (DBS), valproic acid

### 4.2 Longitudinal characterization of AD clinical subtypes

Discoveries of temporal changes and patterns exhibited by representative features within each AD clinical subtype may provide novel insights into disease pathogenesis. By examining the time dimension, the pathogenic circuits specific to each subtype were identified, providing valuable insights for precision medicine. We employed the Gini index to evaluate the importance of features for the samples in the four subtypes at different time points. The detailed calculations and results of the Gini index can be found in Supplementary material B.

**Figure 3:**
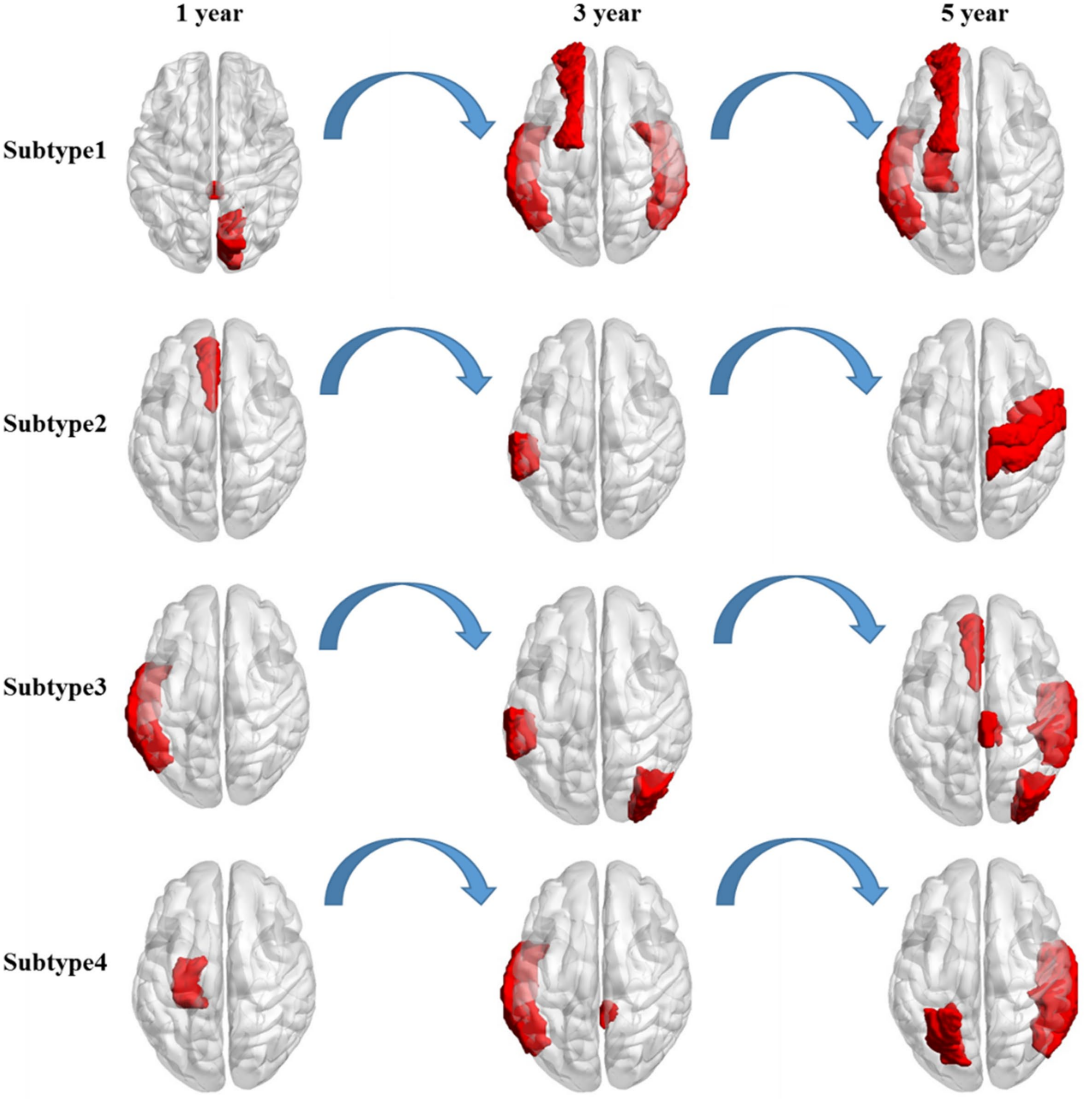
Longitudinal change in the salient-affected brain regions for four clinical subtypes. Each row shows the key brain regions affected by AD in different subtypes over time.

Distinct change patterns of representative features in the time dimension were observed in the four clinical AD subtypes (Figure 3). In the first subtype, AD primarily affects the corpus callosum and right cuneus, followed by the right inferior temporal gyrus, left transverse temporal gyri, and left superior frontal gyrus. Ultimately, it affects the left hippocampus, a critical brain region associated with the onset of AD. In the second subtype, AD initially acts on the left anterior cingulate gyrus, primarily progressing to the left supramarginal gyrus. This eventually affects the right precentral gyrus and right postcentral gyrus, potentially leading to a final stage of general paralysis. In the third subtype, AD initially affects the left temporal lobe, followed by significant involvement of the left supramarginal gyrus and the right occipital lobe. Subsequently, it affects the right superior temporal gyrus, right paracentral lobule, left anterior cingulate gyrus, and right occipital lobe. In the fourth subtype, AD first affects the left entorhinal cortex, followed by the left middle temporal gyrus and right cingulate. Finally, it affects the right transverse temporal gyri, right banks, left parietal cortex, and right middle temporal gyrus. Notably, the pathogenic circuits of the four clinical AD subtypes exhibit distinct patterns. The corpus callosum, cingulate gyrus, temporal lobe, and entorhinal cortex serve as the initial sites for the four subtypes, with subsequent overall brain atrophy occurring over time. These different pathogenic circuits result in diverse clinical manifestations influenced by each patient’s unique physical condition. The discovery of these pathogenic circuits will help clarify the mechanisms underlying the development of AD.

**Figure 4:**
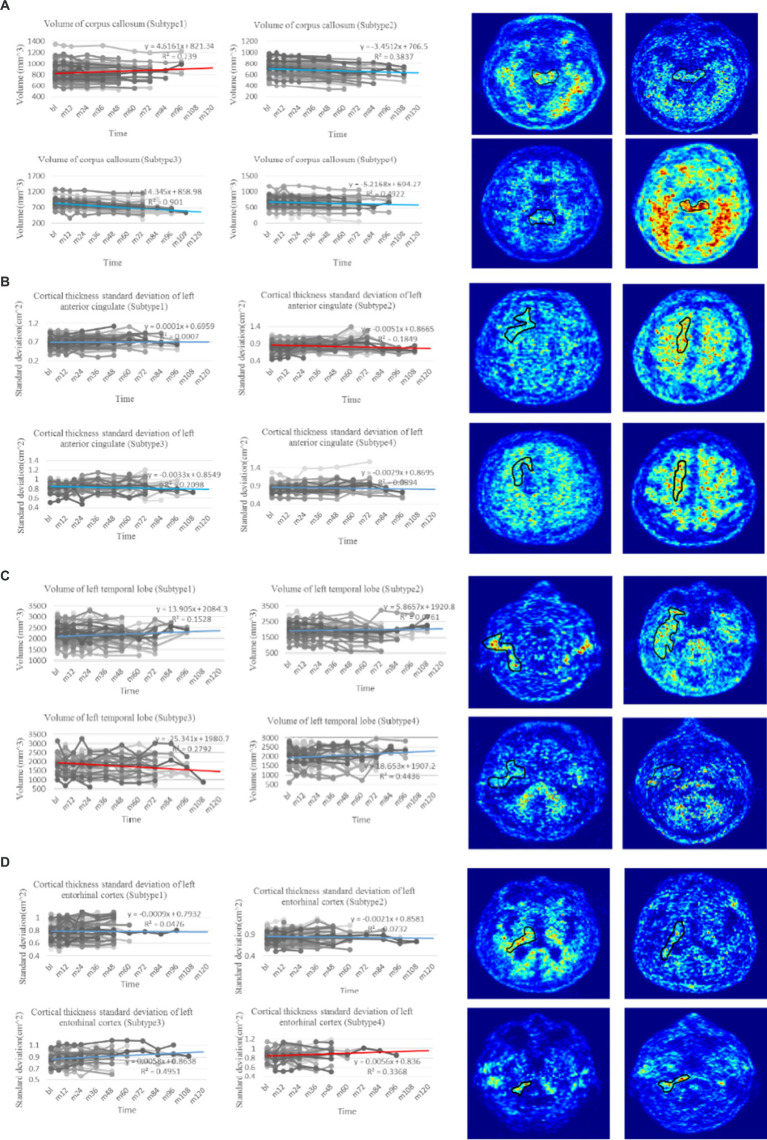
Trend analysis of representative features of four clinical subtypes. **(A)** The line charts on the left are the comparison of change in volume of corpus callosum, which is regarded as the most representative longitudinal feature in the first subtype with *p*-value test ( p≪0.01 ). The PET images on the right show the distribution of plaques in the corpus callosum of four typical patients in the first subtype (18F-AV45). **(B)** The line charts on the left are the comparison of change in cortical thickness standard deviation of left anterior cingulate, which is regarded as the most representative longitudinal feature in the second subtype with *p*-value test ( p≪0.01 ). The PET images on the right show the distribution of plaques in the left anterior cingulate of four typical patients in the second subtype (18F-AV45). **(C)** The line charts on the left are the comparison of change in volume of left temporal lobe, which is regarded as the most representative longitudinal feature in the third subtype with *p*-value test ( p≪0.01 ). The PET images on the right show the distribution of plaques in the left temporal lobe of four typical patients in the third subtype (18F-AV45). **(D)** The line charts on the left are the comparison of change in cortical thickness standard deviation of left entorhinal, which is regarded as the most representative longitudinal feature in the fourth subtype with *p*-value test ( p=0.024 ). The PET images on the right show the distribution of plaques in the left entorhinal of four typical patients in the forth subtype (18F-AV45). The line chart where the red line is located denotes the trend of representative feature in the related subtype, whereas line charts where the blue lines are located denote the trend comparison of the representative feature in the other subtypes.

The National Institute of Aging-Alzheimer’s Association (NIA-AA) proposed a biological classification standard for AD according to the ATN classification system, where A denotes Αβ , T denotes tau protein, and N denotes neurodegeneration (Jack et al., 2018). Although ATN biomarkers provide insights into the early-stage neuropathological processes of AD, they do not rely on clinical diagnostic or phenotypic data, and thus, only reflect the pathophysiological changes of the disease. Despite the widespread use of ATN biomarkers for early detection of AD, they have limitations in explaining the heterogeneity of individual clinical manifestations and predicting the degree of cognitive decline or disease progression. The representative features identified in this study offer a more concise and effective approach than traditional classification methods. Our findings have significant implications for clinical AD research, aiding in identifying individuals at risk of progression and evaluating therapeutic interventions for future cognitive decline. Specifically, the observed changes in the volume (WM Parcellation) of the corpus callosum (Figure 4A), cortical thickness standard deviation of the left anterior cingulate (Figure 4B), volume of the left temporal lobe (Figure 4C), and cortical thickness standard deviation of the left entorhinal (Figure 4D) served as critical longitudinal features for distinguishing the four subtypes. Moreover, confirmed by clinical doctors in Tangdu Hospital, the distribution of plaques (18F-AV45) in the found specific areas of typical patients in the four clinical subtypes are consistent with our conclusion, which further confirms the validity of this discovery. These findings contribute to revealing AD pathogenesis and paving the way for the early diagnosis of the disease.

In this study, we proposed a novel approach called T-cPCA to clinically represent AD by incorporating both the time and feature dimensions of multidimensional AD clinical data. T-cPCA has the advantage of extracting clinical representations from longitudinal AD diagnostic data, considering the evolving nature of AD development over time. To optimize the T-cPCA hyperparameters, we developed an efficient firework algorithm to obtain the optimal hyperparameters that denote the trade-off of natural aging effect elimination. The results of three popular supervised machine-learning algorithms (MLP, RF, and KNN) implemented on ADNI dataset unanimously shows that T-cPCA can make information fusion on time and feature dimensions to improve the efficiency of AD progression prediction models. T-cPCA can enhance our understanding of the disease and improve the accuracy of AD prognosis.

A clustering algorithm called hierarchical clustering was employed to identify AD subtypes for the clinical representation obtained using T-cPCA to cluster the data into four clusters. Subsequently, three popular supervised machine-learning algorithms with consistent parameters were used in these clusters to assess their prediction performance. The results showed varying degrees of improvement in AD progression prediction after clustering, indicating the effectiveness of the identified subtypes and the potential enhancement in the overall prediction accuracy by training prediction models within each subtype. Furthermore, we conducted feature analysis to validate the significance of the four subtypes. Based on the *p*-value test, we identified several longitudinal features that served as distinguishing factors for the four subtypes, including the volume (WM Parcellation) of the corpus callosum, cortical thickness standard deviation of the left anterior cingulate, volume of the left temporal lobe, and cortical thickness standard deviation of the left entorhinal. These findings reveal the specific pathogenic circuits and clinical manifestations of each subtype and offer insights into the early diagnosis of AD.

## 5 Limitations of the study

This is a longitudinal study based on the principle of preserving the richness of data on feature and time dimensions, the analyses lack available data. We are collecting clinical data to extend the existing longitudinal AD dataset together with the Tangdu Hospital. For instance, to predict five-year AD progression, we collected clinical data from participants who have been in continuous research for approximately 10 years, which was challenging. The expanded dataset will provide data support for further research on AD in the future.

## Data availability statement

The data analyzed in this study is subject to the following licenses/restrictions: data used for this study were provided from ADNI studies via data sharing agreements that did not include permission to further share the data. Data from ADNI are available from the ADNI database (adni.loni.usc.edu/) upon registration and compliance with the data usage agreement. Requests to access these datasets should be directed to https://ida.loni.usc.edu/pages/access/studyData.jsp?categoryId=43&subCategoryId=94.

## Ethics statement

The studies involving humans were approved by the Medical Ethics Committee of Xi’an Jiaotong University. The studies were conducted in accordance with the local legislation and institutional requirements. The participants provided their written informed consent to participate in this study. Written informed consent was obtained from the individual(s) for the publication of any potentially identifiable images or data included in this article.

## Author contributions

TH: Formal analysis, Methodology, Writing – original draft. YP: Data curation, Investigation, Writing – review & editing. YD: Visualization, Writing – review & editing. YL: Resources, Visualization, Writing – review & editing. YW: Data curation, Writing – review & editing. WS: Writing – review & editing. LC: Writing – review & editing. QP: Funding acquisition, Supervision, Writing – review & editing.
