# Supplementary material for: Mining Alzheimer’s disease clinical data: reducing effects of natural aging for predicting progression and identifying subtypes
Source: Front Neurosci. 2024 Aug 14;18:1388391. doi: 10.3389/fnins.2024.1388391 (PMC11351280; doi:10.3389/fnins.2024.1388391)
Supplement: Supplementary file 1 [file Table_1.DOCX]

Supplementary Material

# Supplement A. Tensorized contrastive Principal Component Analysis

In this section, we introduce the main contents of Tensorized contrastive Principal Component Analysis and its related works.

## Two-dimensional principal component analysis

The thoughts of Two-dimensional principal component analysis (2DPCA) are the key part of T-cPCA. As the extension of PCA, 2DPCA was firstly proposed by Yang to solve the problem of image reconstruction. After that, 2DPCA has been developed in many fields. Moreover, as the representative extension methods, kernel two-dimensional principal component analysis method and block two-dimensional principal component analysis method were mainly applied in face recognition, palmprint recognition, hyperspectral remote sensing, image dimensionality reduction, moving target detection, etc. The principle of 2DPCA is as follows.

Let $A$ denotes random matrix with $m\times n$ , $X$ denotes the n-dimensional column vector, Then the random matrix $A$ can be projected onto $X$ by the following linear transformation:

$Y=AX$ (1)

Thus, the projection vector $Y$ is obtained, which is called the projection characteristic vector of matrix $A$ . Considering the aim of PCA is to find the direction with the largest variance, it can be naturally deduced that 2DPCA aims to find a projection direction with the largest divergence. The overall scatter matrix of projection samples can be characterized by the trace of the covariance matrix of the projection characteristic vectors. From this perspective, the following calculations are adopted:

$J(X)=tr(S_{x})$ (2)

$S$ denotes the covariance matrix of the projection characteristic vector of the training datasets; $tr(S_{x})$ denotes the trace of $S_{x}$ . When this criterion reaches its maximum value, its physical meaning is to find a projection axis that projects all training samples onto it, so as to maximize the overall scatter matrix of the projected vectors.

The covariance matrix $S_{x}$ of the projection characteristic vector of the training datasets can be expressed as:

$S_{x}=E(Y-EY)(Y-EY)^{T}=E[AX-E(AX)][AX-E(AX)]^{T}=E[(A-EA)X][(A-EA)X]^{T}$ (3)

Define matrix:

$G=E[(A-EA)^{T}(A-EA)]$ (4)

The matrix $G$ , which is nonnegative definite matrix, is called the scatter matrix of random matrix $A$. Assuming that the training samples are $A_{j}(j=1,2,...,M)$, and the average matrix of all training samples is $\overline{A}$, $G$ can be written as:

$G=\frac{1}{M}\sum_{j=1}^{M} (A_{j}-\overline{A})^{T}(A_{j}-\overline{A})$ (5)

Formula (2) can be simplified as:

$J(X)=X^{T}GX$ (6)

$X$ is called the optimal projection axis which maximizes $J(X)$.

## Contrastive principal component analysis

The thoughts of contrastive principal component analysis (cPCA) are another key part of T-cPCA algorithm. The cPCA was developed to find contrastive principal components (cPCs) that maximize variance in the target dataset and minimize the variance in the background dataset. The development of cPCA was motivated by the need to detect and visualize variation in the data deemed most relevant for the scientific question of interest. Given a target dataset believed to contain signal of interest and a similar background dataset believed to comprise only noise, the cPCA algorithm obtains a subspace of the target data that contains the variation absent from the background data. The principle of cPCA is as follows.

For ease of description, $\{X_{i}\}_{i=1}^{n}$ is denoted as the target dataset and $\{Y_{j}\}_{j=1}^{m}$ is denoted as the background dataset. We assumed that the datasets have been standardized and their covariance matrices are expressed by $C_{X}$ and $C_{Y}$ respectively. The variances in the target data and background data can be defined as follows:

Target variance: $\text{ }\lambda_{X}(v)=v^{T}C_{X}v$ (7)

Background variance: $\text{ }\lambda_{Y}(v)=v^{T}C_{Y}v$ (8)

The purpose of contrastive principal component analysis is to find the vector $v$ , for which has a large variance in the target data and a small variance in the background data. The specific can be expressed by the following formula:

$\mathop{\mathrm{argmax}}\limits_{v\in R_{unit}^{d}} (\text{ }\lambda_{X}(v)-\alpha\text{ }\lambda_{Y}(v))$ (9)

The comparison parameter $\alpha\in[0,\infty]$.

## T-cPCA

The specific algorithm of T-cPCA is as follows (Algorithm 1):

| **Algorithm 1** T-cPCA For Given $\alpha_{feature}$ |
| --- |
| **Inputs:** Target dataset and background dataset: $S^{Tar}$, $S^{Back}$; contrast parameters, $\alpha_{feature}$ ; the number of components, $m_{feature}$, $m_{time}$.  **Step1:** Normalize the data $S^{Tar}$, $S^{Back}$.  **Step2:** Calculate the covariance matrices of target data and background data in feature dimension according to *(10)* and *(11)* respectively to obtain $C_{Tar}^{feature}$ and $C_{Back}^{feature}$.  **Step3:** Perform eigenvalue decomposition on the contrast covariance matrix. $C^{feature}=C_{Tar}^{feature}-\alpha_{feature}C_{Back}^{feature}$   **Step4:** Compute the the subspace $M^{feature}\in R^{k\times m_{feature}}$ spanned by the top $m_{feature}$ eigenvectors of $C^{feature}$ **Step5:** Calculate the covariance matrices of target data and background data in time dimension according to *(13)* and *(14)* respectively to obtain $C_{Tar}^{time}$ and $C_{Back}^{time}$.  **Step6:** Perform eigenvalue decomposition on the contrast covariance matrix. $C^{time}=C_{Tar}^{time}-\alpha_{time}C_{Back}^{time}$   **Step7:** Compute the the subspace $M^{time}\in R^{p\times m_{time}}$spanned by the top $m_{time}$eigenvectors of $C^{time}$.  **Return:** the subspace $M^{feature}$ , $M^{time}$. |

## Selection of Comparison parameters

| **Algorithm 2** Fireworks Algorithm For the Selection of $\alpha_{feature}$, $\alpha_{time}$ |
| --- |
| **Inputs:** Target and background data: $S^{Tar}$, $S^{Back}$; the number of components $m_{feature}$,$m_{time}$, the number of initial dataset $q_{feature}$, the number of initial dataset $q_{time}$, the maximum radius of explosion $\widehat{A}$ , offset coefficient $\varepsilon$, the number of generated fireworks $r_{1}$ and $r_{2}$, affinity function $f$, update parameter $k$.  While literation:  **for** each , $\alpha_{time}=null$ **do**  Compute the subspace set $\{M_{i}^{feature}\}_{i=1,2,...,q_{feature}}$ using **Algorithm 1.**  Compute the value of affinity function set $\{f_{M_{i}^{feature}}(S^{Tar})\}$ , where $f$ is chosen as contrastive loss.  **end for**  **for** each **do**  Compute the maximum radius .  Select the corresponding to the top $k$ smallest $f_{M_{i}^{feature}}(S^{Tar})$ and keep it in the set and remove other.  **for** each **do**  Take as the center and its corresponding $A_{i}$ as the radius to randomly generate $r_{1}$ samples and update . Randomly generate $r_{2}$ samples at the range $(0,1000)$ and update .  **end for**  **If** $f_{\min}$ converges:    Break  **end if**  **Similarly, we can get** $\alpha_{time}$ $\alpha$ **Return:** $\alpha_{feature}$, $\alpha_{time}$ |

The comparison parameter $\alpha$ denotes the trade-off between maximizing the target variance and minimizing the background variance. When $\alpha=0$ , The projection direction selected by cPCA only maximizes the target variance. At this time, the model degenerates to PCA for the target dataset. While $\alpha$ gradually increases, the effect of background variance becomes greater. In this section, a advanced fireworks algorithm is proposed to make selection of parameter . The specific algorithm is shown in Algorithm 2.


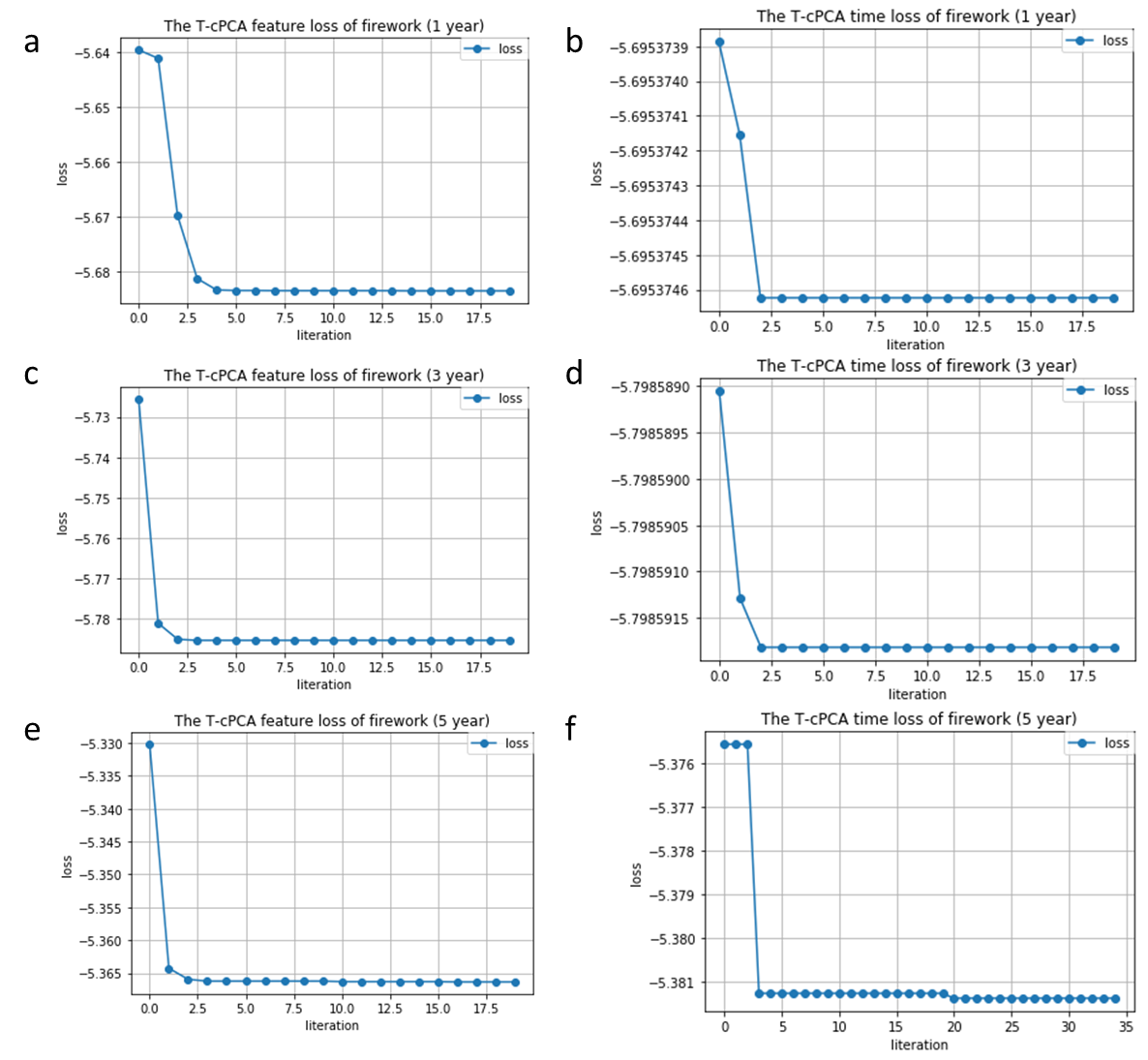


**Supplementary Figure 2.** The convergence of fireworks algorithm on feature dimension and time dimension. (a) The convergence of fireworks algorithm on feature dimension in the task of 1-year AD progression prediction. (b) The convergence of fireworks algorithm on time dimension in the task of 1-year AD progression prediction. (c) The convergence of fireworks algorithm on feature dimension in the task of 3-year AD progression prediction. (d) The convergence of fireworks algorithm on time dimension in the task of 3-year AD progression prediction. (e) The convergence of fireworks algorithm on feature dimension in the task of 5-year AD progression prediction. (f) The convergence of fireworks algorithm on time dimension in the task of 5-year AD progression prediction.

For time dimension and feature dimension, the loss of firework can fast converge to a fixed value (Figure.2). The experimental results show that the fireworks algorithm can find the optimal value of hyperparameters in a large range quickly and accurately.

## The technical details about the machine learning algorithms

Three popular supervised machine-learning algorithms (Multilayer Perceptron, Random Forest, and K-Nearest Neighbors algorithm) are applied to verify the effectiveness of T-cPCA. The technical details of these machine learning algorithms are introduced in this section.

### Multilayer Perceptron

Multilayer Perceptron (MLP) is a deep learning model based on Feedforward Neural Network. Each neuron layer of a multi-layer perceptron is composed of many neurons, where the input layer receives input features, the output layer provides the final prediction results, and the hidden layer in the middle is used to extract features and perform nonlinear transformations. Each neuron receives the output of the previous layer and performs weighted sum and activation function operations to obtain the output of the current layer. Through continuous iterative training, multi-layer perceptron can automatically learn the complex relationships between input features and make prediction. In the experiment, we utilized sklearn.neural_network.MLPClassifier to make classifier. The number of MLPlayers is chosen as 3, with the number of neurons in each layer being 60, 80, and 40, respectively

### Random Forest

Random forest refers to a classifier that uses multiple decision trees to train and predict in different tasks. It contains multiple decision tree classifiers, and its output is determined by the output of individual trees. Random forest is a flexible and easy-to-use machine learning algorithm which can achieve good results in most cases without hyperparameter tuning. The random forest integrates all classification voting results, specifying the category with the highest number of votes as the final output. In the experiment, we utilized sklearn.ensemble.RandomForestClassifier to make classification. The number of decision trees was selected as 20.

### K-Nearest Neighbors

The K-Nearest Neighbor (KNN) classification algorithm is one of the efficient methods in data mining and classification technology. It is a well-known pattern recognition statistical method and holds a significant position in machine learning classification algorithms. It is a relatively mature method in theory, we utilized sklearn.neighbors.KNeighborsClassifier to make classification. The K was selected as 4.

# Supplement B. AD clinical subtypes identification

In this section, we introduce the details of clinical AD subtypes identification.

## Hierarchical clustering algorithm

With the advantage of less restrictions and better clustering quality, hierarchical clustering algorithm has been widly used in protein sequence data analysis, gene expression data analysis, financial time series analysis, etc. According to the top-down or bottom-up principle of hierarchical decomposition, hierarchical clustering can be divided into divisive hierarchical clustering strategy and agglomerative hierarchical clustering strategy. For agglomerative hierarchical clustering strategy, the aggregation process of clustering should follow some principles, which means that whether to continue merging the current two clusters is based on the connectivity measurement. The measurement has the following three methods called ‘Ward’, ‘Maximum’ and ‘Average’. Here agglomerative hierarchical clustering strategy is used to identify AD clinical subtypes with ‘Maximum’ measurement. In order to determine the number of cluster, silhouette score is used to evaluate the clustering result. The higher the silhouette score, the better the clustering performance. silhouette score is composed of two scores named $a$ and $b$. $a$ denotes the average distance between the sample and all other samples in the same cluster, and $b$ denotes the average distance between the sample and all other samples in its nearest cluster. The silhouette score is defined as followed:

$Silhouette\_score=\frac{b-a}{\max(a,b)}$ (17)

After T-cPCA representation, the AD clinical data are clusterd to identify the subtypes. We find that while the cluster number is chosen as 4, the silhouette score reaches the peak (Figure 3). Thus the cluster number is chosen as 4 in subsequent analysis.

**Supplementary Figure 3.** The relationship between silhouette score and the cluster number.

## Comparison of the importance of top 10 features based on gini index in different subtypes

**Supplementary Table 1.** The importance of top 10 features based on gini index in different subtypes

| **FLDNAME** | **Feature** | **Importance** |
| --- | --- | --- |
| **Subtype1** |  |  |
| CTX_LH_UNKNOWN_UCBERKELEYAV45_10_17_16 | ctx-lh-unknown | 0.024631288 |
| **ST2SV_UCSFFSX_11_02_15_UCSFFSX51_08_01_16** | **Volume (WM Parcellation) of CorpusCallosumAnterior** | **0.027695804** |
| PTETHCAT | Ethnicity | 0.023856404 |
| PTRACCAT | Race | 0.020149212 |
| PTMARRY | Marital status at baseline | 0.022061101 |
| ST93SA_UCSFFSL_02_01_16_UCSFFSL51ALL_08_01_16 | Surface Area of RightIsthmusCingulate | 0.018975784 |
| ST82TA_UCSFFSL_02_01_16_UCSFFSL51ALL_08_01_16 | Cortical Thickness Average of RightCuneus | 0.021686002 |
| ST24SA_UCSFFSL_02_01_16_UCSFFSL51ALL_08_01_16 | Surface Area of LeftEntorhinal | 0.016262722 |
| ST6SV_UCSFFSL_02_01_16_UCSFFSL51ALL_08_01_16 | Volume (WM Parcellation) of CorpusCallosumPosterior | 0.016983286 |
| CTX_LH_BANKSSTS_UCBERKELEYAV45_10_17_16 | ctx-lh-bankssts | 0.014663465 |
| **Subtype2** |  |  |
| ST99TS_UCSFFSL_02_01_16_UCSFFSL51ALL_08_01_16 | Cortical Thickness Standard Deviation of RightMiddleTemporal | 0.014201174 |
| ST49TS_UCSFFSX_11_02_15_UCSFFSX51_08_01_16 | Cortical Thickness Standard Deviation of LeftPostcentral | 0.014132402 |
| PTETHCAT | Ethnicity | 0.018704251 |
| PTMARRY | Marital status at baseline | 0.014605191 |
| ST59SA_UCSFFSX_11_02_15_UCSFFSX51_08_01_16 | Surface Area of LeftSupramarginal | 0.017338316 |
| **ST14TS_UCSFFSX_11_02_15_UCSFFSX51_08_01_16** | **Cortical Thickness Standard Deviation of LeftCaudalAnteriorCingulate** | **0.023799219** |
| ST13TA_UCSFFSL_02_01_16_UCSFFSL51ALL_08_01_16 | Cortical Thickness Average of LeftBankssts | 0.014043407 |
| ST59SA_UCSFFSL_02_01_16_UCSFFSL51ALL_08_01_16 | Surface Area of LeftSupramarginal | 0.016356058 |
| ST32TS_UCSFFSX_11_02_15_UCSFFSX51_08_01_16 | Cortical Thickness Standard Deviation of LeftInferiorTemporal | 0.010680636 |
| ST25CV_UCSFFSX_11_02_15_UCSFFSX51_08_01_16 | Volume (Cortical Parcellation) of LeftFrontalPole | 0.015190726 |
| **Subtype3** |  |  |
| PTRACCAT | Race | 0.01174049 |
| PTMARRY | Marital status at baseline | 0.012935934 |
| ST59TA_UCSFFSX_11_02_15_UCSFFSX51_08_01_16 | Cortical Thickness Average of LeftSupramarginal | 0.014131133 |
| ST91TA_UCSFFSL_02_01_16_UCSFFSL51ALL_08_01_16 | Cortical Thickness Average of RightInferiorTemporal | 0.016384517 |
| **ST60CV_UCSFFSX_11_02_15_UCSFFSX51_08_01_16** | **Volume (Cortical Parcellation) of LeftTemporalPole** | **0.024983674** |
| ST94SA_UCSFFSX_11_02_15_UCSFFSX51_08_01_16 | Surface Area of RightLateralOccipital | 0.009871284 |
| ST24TA_UCSFFSX_11_02_15_UCSFFSX51_08_01_16 | Cortical Thickness Average of LeftEntorhinal | 0.009413142 |
| ST56CV_UCSFFSX_11_02_15_UCSFFSX51_08_01_16 | Volume (Cortical Parcellation) of LeftSuperiorFrontal | 0.013034478 |
| ST48TA_UCSFFSX_11_02_15_UCSFFSX51_08_01_16 | Cortical Thickness Average of LeftPericalcarine | 0.011819022 |
| ST48TS_UCSFFSX_11_02_15_UCSFFSX51_08_01_16 | Cortical Thickness Standard Deviation of LeftPericalcarine | 0.010073927 |
| **Subtype4** |  |  |
| PTGENDER | Sex | 0.013276626 |
| PTETHCAT | Ethnicity | 0.014165182 |
| PTMARRY | Marital status at baseline | 0.013476902 |
| ST110CV_UCSFFSL_02_01_16_UCSFFSL51ALL_08_01_16 | Volume (Cortical Parcellation) of RightPrecentral | 0.013052765 |
| ST98SA_UCSFFSL_02_01_16_UCSFFSL51ALL_08_01_16 | Surface Area of RightMedialOrbitofrontal | 0.017130914 |
| ST32TS_UCSFFSL_02_01_16_UCSFFSL51ALL_08_01_16 | Cortical Thickness Standard Deviation of LeftInferiorTemporal | 0.013885567 |
| **ST24TS_UCSFFSL_02_01_16_UCSFFSL51ALL_08_01_16** | **Cortical Thickness Standard Deviation of LeftEntorhinal** | **0.055570706** |
| ST106CV_UCSFFSX_11_02_15_UCSFFSX51_08_01_16 | Volume (Cortical Parcellation) of RightParsTriangularis | 0.015454383 |
| ST38TA_UCSFFSX_11_02_15_UCSFFSX51_08_01_16 | Cortical Thickness Average of LeftLingual | 0.015448841 |
| ST115TS_UCSFFSX_11_02_15_UCSFFSX51_08_01_16 | Cortical Thickness Standard Deviation of RightSuperiorFrontal | 0.012601503 |
